# Self-Assembled Micelles Composed of Doxorubicin Conjugated Y-Shaped PEG-Poly(glutamic acid)_2_ Copolymers via Hydrazone Linkers

**DOI:** 10.3390/molecules190811915

**Published:** 2014-08-11

**Authors:** Bowen Sui, Hui Xu, Jian Jin, Jingxin Gou, Jingshuo Liu, Xing Tang, Yu Zhang, Jinghua Xu, Hongfeng Zhang, Xiangqun Jin

**Affiliations:** 1 Department of Pharmaceutics, College of Pharmacy Sciences, Jilin University, Changchun 130021, Jilin, China; 2 Department of Pharmaceutics, Shenyang Pharmaceutical University, Shenyang 110016, China; 3 School of Life Science and Biopharmaceutics, Shenyang Pharmaceutical University, Shenyang 110016, Liaoning, China

**Keywords:** miktoarm-star copolymer, conjugate, doxorubicin, pH-responsive drug release, drug delivery

## Abstract

In this work, micelles composed of doxorubicin-conjugated Y-shaped copolymers (YMs) linked via an acid-labile linker were constructed. Y-shaped copolymers of mPEG-b-poly(glutamate-hydrazone-doxorubicin)_2_ and linear copolymers of mPEG-b-poly(glutamate-hydrazone-doxorubicin) were synthesized and characterized. Particle size, size distribution, morphology, drug loading content (DLC) and drug release of the micelles were determined. Alterations in size and DLC of the micelles could be achieved by varying the hydrophobic block lengths. Moreover, at fixed DLCs, YMs showed a smaller diameter than micelles composed of linear copolymers (LMs). Also, all prepared micelles showed sustained release behaviors under physiological conditions over 72 h. DOX loaded in YMs was released more completely, with 30% more drug released in acid. The anti-tumor efficacy of the micelles against HeLa cells was evaluated by MTT assays, and YMs exhibited stronger cytotoxic effects than LMs in a dose- and time-dependent manner. Cellular uptake studied by CLSM indicated that YMs and LMs were readily taken up by HeLa cells. According to the results of this study, doxorubicin-conjugated Y-shaped PEG-(polypeptide)_2_ copolymers showed advantages over linear copolymers, like assembling into smaller nanoparticles, faster drug release in acid, which may correspond to higher cellular uptake and enhanced extracellular/intracellular drug release, indicating their potential in constructing nano-sized drug delivery systems.

## 1. Introduction

Benefitting from the advances in polymer science, more and more novel synthetic polymers are being used in the construction of nano-scale drug delivery systems (DDS) that target tumors by their enhanced permeation and retention (EPR) effects [1]. Simple alterations in the morphology, size, release profile or drug-loading content of nano-carriers can be readily achieved by modifying parameters like the generations of dendrimers [2,3], grafting percentage of graft copolymers [4,5] and the conformation of the blocks [6,7]. Recently, miktoarm-star copolymers with branches at the junction point of the linear copolymers have aroused increasing interest [8,9,10,11]. These non-linear copolymers exhibited unique self-assembly behaviors believed to be based on the conformational entropy reduction caused by the introduction of branches at the junction point [10,12]. The architecture dependent self-assemble of these polymers affords conventional and non-conventional nano-structures like micelles [8,10,11], polymersomes [9,13], multicompartment micelles [14] and wormlike micelles [15]. More recently, polyamino acids were used to build a variety of polymers due to their high biocompatibility. Also, secondary structures like α-helix and β-sheets adopted by natural or synthetic polyamino acids endowed them advantages in terms of stability and processability [10]. The application of miktoarm-star copolymers for drug delivery has been reviewed by Soliman *et al*. [16]. For example, PNIPAM-b-poly(undecylenic acid)_2_ was synthesized by Li *et al.* [17] to construct prednisone acetate loaded thermal-responsive micelles, which showed a temperature-dependent sustained release. However, the micellar stability might be limited due to the flexibility of the all-carbon backbone of the polymer, as reflected by a higher CAC value, while the secondary structure adopted by poly(amino acids) reduced chain mobility, thereby increasing the micellar stability to some extent. Another AB_2_ type copolymer (mPEG-b-PBLG_2_) was synthesized by Li *et al*. [8], and the tamoxifen loaded micelles also exhibited a sustained release pattern.

For micellar systems composed of AB type copolymers, increasing the length of the hydrophobic chain is a direct way to increase the DLC and reduce the drug release rate. However, micelles with longer hydrophobic chain lengths might be less stable due to the reduced hydrophilic surface coverage [8,18,19]. According to the above mentioned self-assembling properties of miktoarm-star copolymers, it seems like that the hydrophobic chains of miktoarm-star copolymers could be shorter than the AB type counterparts with similar degrees of polymerization, which means a better stability and a smaller diameter of the micelles. Likewise, for miktoarm-star copolymers with similar single hydrophobic chain lengths, more cargo could be loaded into the micelles, so some new traits might be possible by constructing micelles with these branched polymers.

Doxorubicin (DOX) is a widely used antitumor drug that has been involved in a great deal of research into the development of DDSs. The reason for its popularity may be due to its unique physical-chemical properties. The salt form of DOX (DOX·HCl) is water-soluble and can be incorporated into liposomes [20] and polymersomes [21], and it could form aggregates with anionic polymers through electrostatic interactions [22]. When neutralized, DOX becomes insoluble in water and can be loaded into the hydrophobic cores of micelles [23]. Moreover, an acid-labile hydrazone linker could be introduced to achieve a pH-responsive release [24]. Also, for DDSs carrying DOX or other chemotherapeutic agents, the drug loading content (DLC) is a key parameter that is closely related to the anti-tumor efficacy and even multidrug resistance induction. This indicates that further increasing drug loading while maintaining a small particle size is a task that still remains to be accomplished.

**Scheme 1:**
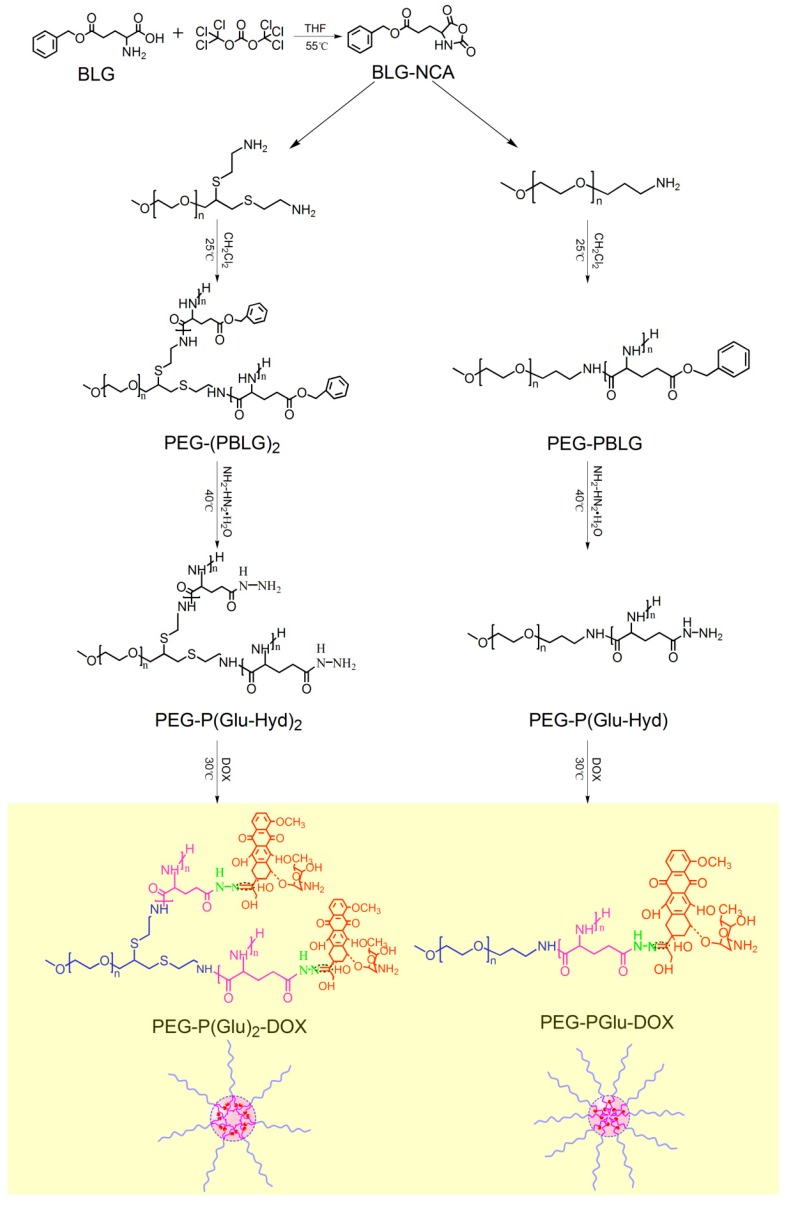
Synthetic routes of linear or Y-shaped PEG-poly(Glu-Hyd-DOX) polymers and formation of micelles.

In this study, Y-shaped miktoarm-star copolymers of α-methoxypoly(ethylene glycol)-b-poly (glutamate-hydrazone-DOX)_2_ (mPEG-b-p(Glu-Hyd-DOX)_2_) were synthesized (Scheme 1). In addition, AB type linear copolymers mPEG-b-poly(Glu-Hyd-DOX) were also synthesized. Both mPEG and poly (glutamic acid) are biocompatible and have been widely used in various studies. DOX was conjugated to the polymers through an established hydrazone-linker [25] to achieve a stimuli-responsive release and less leakage into the bloodstream. In order to confirm the above hypotheses, micelles composed of these Y-shaped and linear copolymers were prepared and their size, DLC, *in vitro* release and cytotoxicity were investigated.

## 2. Results and Discussion

### 2.1. Synthesis and Characterization of Polymers

The ring-opening polymerization (ROP) of amino acid NCAs is a commonly employed method in the facile synthesis of polyamino acids with controllable degrees of polymerization and polydispersity indices (PDI) [7]. Y-shaped miktoarm-star mPEG-(PBLG)_2_ copolymers with different molecular weights were synthesized via ring-opening polymerization of γ-benzyl-l-glutamate-*N*-carboxy anhydride (BLG-NCA) using α-methoxy-poly(ethylene glycol) with two primary amino groups at the ω-terminal as a macromolecular initiator [8]. DOX was conjugated to the polymers via an acid-labile hydrazone linker with hydrazide groups obtained from EAE aminolysis of the PBLG block. The synthetic route is shown in Scheme 1.

**Figure 1:**
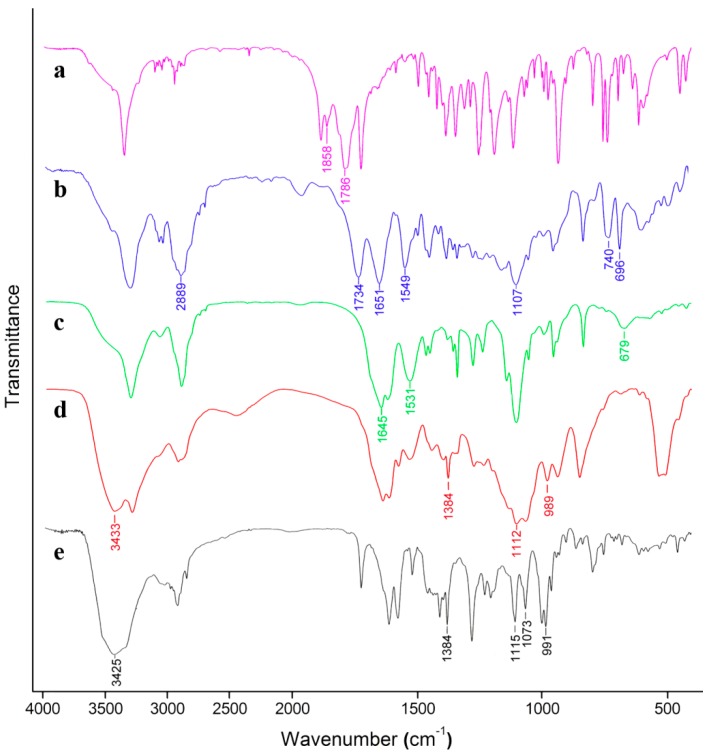
Fourier transform infrared spectroscopy spectra of: BLG-NCA (**a**); PEG-(PBLG)_2_ (**b**); PEG-poly(Glu-Hyd)_2_ (**c**); PEG-poly(Glu)_2_-DOX polymers (**d**) and DOX·HCl (**e**).

The chemical structures of the products were confirmed by FTIR and ^1^H-NMR. Figure 1 shows the FTIR spectra of BLG-NCA, mPEG-(PBLG)_2_, mPEG-p(Glu-Hyd)_2_ and mPEG-p(Glu)_2_-DOX polymers. The strong absorptions at 1858 cm^−1^ and 1786 cm^−1^ in Figure 1a were the characteristic peaks of the –CO-O-CO- end group of BLG-NCA, which had disappeared completely in the mPEG-(PBLG)_2_ FTIR spectrum in Figure 1b. This indicated the complete transformation of BLA-NCA. The disappearance, together with the emergence of an amide I and amide II bands at 1651 cm^−1^ and 1549 cm^−1^ was due to the formation of amide groups in mPEG-(PBLG)_2_, and the marked peak at 1107 cm^−1^ was attributed to the C-O-C stretching vibration and the peak at 2889 cm^−1^ proved the incorporation of mPEG-S-(CH_2_CH_2_NH_2_)_2_. The disappearance of the stretch vibration of the ester C=O (1734 cm^−1^) and the reduced absorbance at 696 cm^−1^ and 740 cm^−1^ (characteristic peaks of mono-substituted phenyl group) as well as the appearance of amide band 1645 cm^−1^ and 1531 cm^−1^ all indicated the conjugation of hydrazide (Figure 1c). The conjugation of DOX was confirmed by the appearance of peaks at 989 cm^−1^, 1,384 cm^−1^ and 3,433 cm^−1^ and the widening of the peak at 1112 cm^−1^ in Figure 1c‒e.

**Figure 2:**
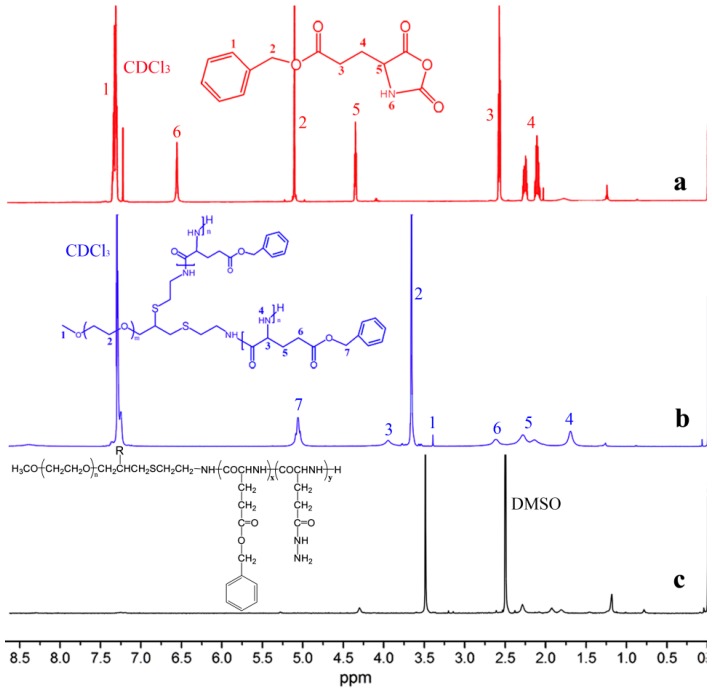
^1^H-NMR spectra of BLG-NCA (**a**); PEG-(PBLG)_2_ (**b**) copolymer in CDCl_3_ and PEG-poly(Glu-Hyd)_2_ (**c**) polymer in CF_3_COOD/DMSO-*d_6_*.

The ^1^H-NMR spectra of the BLG-NCA and polymers in CDCl_3_ or CF_3_COOD/DMSO-*d_6_* are shown in Figure 2. In Figure 2a, the smooth baseline in the spectrum of BLG-NCA confirmed its high purity since this monomer is relatively sensitive to humidity and the quality of the polymer is dependent on the purity of the monomers. The NMR spectrum of the mPEG-(PBLG)_2_ copolymer in Figure 2b exhibited characteristic peaks at 3.39 ppm (-C**H**_3_O-) and 3.66 ppm (-C**H**_2_-C**H**_2_-O-), which were assigned to the proton of the MPEG block. Peaks at 5.06 ppm (-C**H**_2_-C_6_H_5_), 3.95 ppm (-C**H**-CO-NH-), 2.63 ppm (-CH_2_-C**H**_2_-COO-), and 2.15-2.29 ppm (-C**H**_2_-CH_2_-COO-) were assigned to the protons of the PBLG unit. The degree of polymerization was calculated from the signals at δ3.66 ppm and δ5.06 ppm as they appeared with good reproducibility and little sign of interference. As shown in Figure 2c, approximately all of the benzyl esters were replaced by hydrazide groups as the signals of the -CH_2_- group (δ5.06) were nearly unnoticeable. The NMR spectra of benzyl ester protected polymers and hydrazinolyzed polymers are shown in Figure S1and Figure S2.

The molecular weights of the PEG-PBLG or PEG-PBLG_2_ copolymers calculated by ^1^H-NMR are listed in Table 1. The initiators employed showed an initiating efficiency of no less than 0.8, which means a controllable constitution of polymers could be achieved by varying the feed ratios of the initiators and monomers. As verified by the GPC in Figure S3, the polymers synthesized displayed unimodal molecular weight distributions with little impurities, and a constant PDI less than 1.20 was also obtained (Table 1). The retention time of PEG_5k_-(PBLG_5.7k_)_2_ is 0.33 min shorter (22.98 min *vs.* 22.65 min) than that of its linear counterpart with a similar molecular weight. This can be ascribed to the higher molecular volume of the miktoarm star copolymers.

**Table 1:** Compositions and some characteristic properties of hybrid polypeptide copolymers.

Copolymer ^a^	Feed molar ratio of [I]/[N] ^b^	M_n_ of copolymer ^c^ (Da)	DP ^d^	Initiator efficiency ^e^	Polydispersity ^f^ (M_W_/M_n_)
PEG_5k_-PBLG_5.7k_	1/30	10694	26	0.87	1.07
PEG_5k_-PBLG_13.6k_	1/70	18578	62	0.89	1.19
PEG_5k_-(PBLG_5.7k_)_2_	1/65	16388	52	0.80	1.11
PEG_5k_-(PBLG_6.8k_)_2_	1/75	18578	62	0.83	1.11

Notes: ^a^ The subscripts indicate the mean Mn of each block; ^b^ [I]/[N] refers to the molar ratio of initiator (MPEG-NH_2_ or MPEG-S-(CH_2_CH_2_NH_2_)_2_) to monomer (BLG-NCA); ^c^ determined by ^1^H-NMR analysis; ^d^ refers to the degree of polymerization and calculated by ^1^H-NMR; ^e^ calculated by DP_calculated_/DP_targeted_; ^f^ measured by GPC.

**Figure 3:**
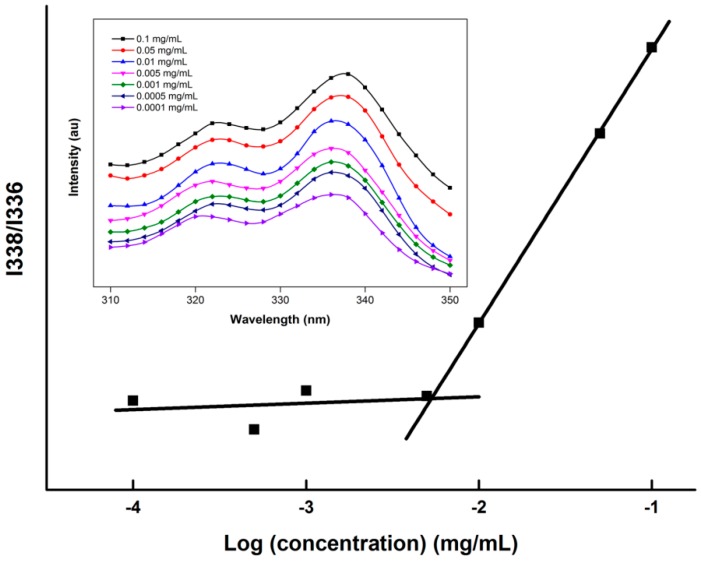
The representative plots of I338/I336 *versus* log C of PEG-p(Glu)_2_-DOX polymer. The excitation spectra of polymer solution with different concentrations in the presence of pyrene are shown in the inset.

The anti-dilution ability which is usually presented as the CAC of polymeric micelles is a key parameter that determines the micellar performance *in vivo*. The CAC of the drug-conjugated copolymers was characterized by steady-state fluorescent probe studies. Pyrene was chosen as the fluorescent probe because of its photophysical characteristics and other properties [26]. Pyrene will preferentially partition into hydrophobic microdomains with a concurrent change in the photophysical properties of the molecule. The maximum excitation wavelength of pyrene increased from 336 nm to 338 nm *versus* the polymer concentration as shown in Figure 3. And we found DOX showed extremely low excitation intensity in the range of 300 nm to 350 nm. Therefore, the fluorescence intensity ratio at 338 and 336 nm (*I*_338_/*I*_336_) of pyrene *versus* the logarithm of the polymer concentration was plotted from the excitation spectra (Figure 3 inset). The CAC of the polymer was calculated to be the corresponding concentration at the inflection point of the plot representing the formation of micelles. The calculated CACs of the copolymers are shown in Table 2 and a slightly lower CAC of the Y-shaped copolymers was observed. This indicated a better thermodynamic stability of YMs compared with LMs. The trend of reduced CAC values with increased hydrophobic block lengths is in accordance with other’s data [7]. However, we noticed that the CAC values of the Y-shaped copolymers differed from those of other studies. Both Yang’s [27] and Maglio’s [28] groups compared the CAC values between linear and Y-shaped copolymers with similar molecular weights, and a higher CAC of the Y-shaped copolymers was found, which is not in accordance with our results. This might be caused by differences in the physico-chemical properties of the hydrophobic blocks. Poly(solketal acrlate) and poly(ε-caprolactone) were employed as hydrophobic blocks in studies by Nie and Quaglia, respectively, and the Y-shaped copolymers in this CAC test are mPEG-PG_2_Ds, so the discrepancy in CAC might be related to the secondary structures adopted by PBLGs and another study involving the same hydrophobic blocks showed identical trends in CAC changes [29].

**Table 2:** Characteristics of different doxorubicin-conjugated micelles.

Sample	Polymer	Size (nm)	Polydispersity index	DLC (%)	CAC (mg/L)
PEG-PGD_I_	PEG-P(Glu)_26_-DOX	149.9 ± 3.7	0.081 ± 0.023	9.92 ± 0.25	9.0
PEG-PGD_II_	PEG-P(Glu)_62_-DOX	231.4 ± 8.3	0.229 ± 0.061	18.8 ± 0.18	4.2
PEG-PG_2_D_I_	PEG-P(Glu_26_)_2_-DOX	141.3 ± 5.2	0.222 ± 0.042	16.2 ± 0.12	5.4
PEG-PG_2_D_II_	PEG-P(Glu_31_)_2_-DOX	165.6 ± 2.4	0.116 ± 0.035	18.2 ± 0.45	3.8

Notes: Data represent mean ± standard deviation (n = 3).

### 2.2. Preparation of DOX-Conjugated Polymer Micelles

**Figure 4:**
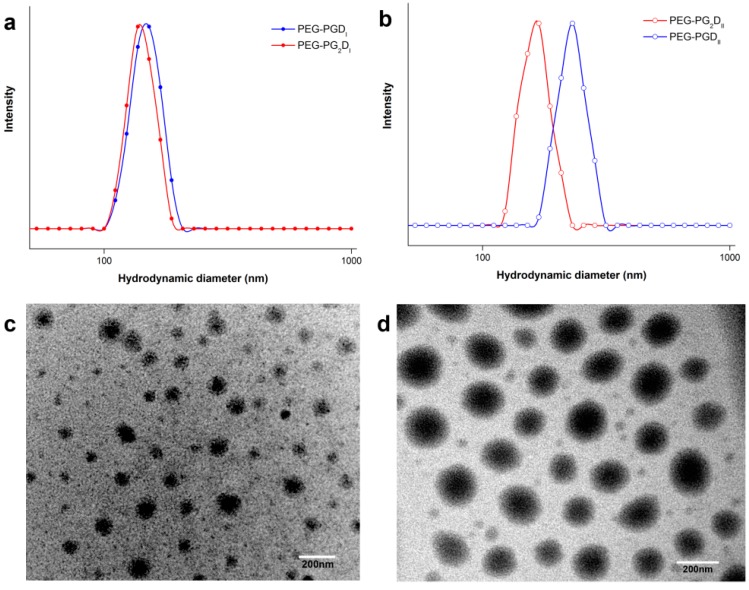
Particle size characterization of prepared micelles. The dynamic laser light scattering analysis of PEG-PGD and Y-shaped PEG-PG_2_D micelles (**a**) or (**b**), Transmission electron microscopy images of PEG-PG_2_D_II_ (**c**) and PEG-PGD_II_ (**d**) micelles.

DOX-conjugated micelles were prepared by dialysis. PBS (pH 7.4, 10 mM) and deionized (DI) water were used as the dispersion medium. In this manner, DOX-conjugated micelles based on PEG hybrid polypeptides were prepared. The particle sizes, size distributions and DLCs of all prepared micelles are shown in Table 2. According to the results, the diameter data further confirmed that the particle sizes are determined by the lengths of the hydrophobic blocks. The morphologies of the prepared micelles were examined by TEM (Figure 4c,d) and the micellar core-shell structure was clearly presented.

Also, when judged in combination with DLC, the advantage of YMs was clear: for micelles with similar diameters (PEG-PGD_I_ and PEG-PG_2_D_I_ in Figure 4(a)), the DLC of PEG-PG_2_D_I_ is nearly twice as that of PEG-PGD_I_ (16.2% *vs.* 9.92%) as the Y-shaped copolymers provided more sites for DOX conjugation at poly glutamic acid block lengths similar to that of the linear copolymers. However, due to the steric hindrance caused by the loaded DOX, a doubled DLC was not achieved. On the other hand, for micelles with similar DLCs (PEG-PGD_II_ and PEG-PG_2_D_II_), the diameters of the YMs were significantly smaller (231.4 nm *vs.* 165.6 nm, Figure 4b) which indicated better accumulation could be achieved as smaller micelles could more readily pass through tumor vessels [1] and penetrate into tumor tissues [30]. This provided a method to use the limited micellar core in a more efficient manner. The determined results for the particle size and DLC are in accordance with our previously stated hypothesis.

### 2.3. In Vitro Drug Release Studies

**Figure 5:**
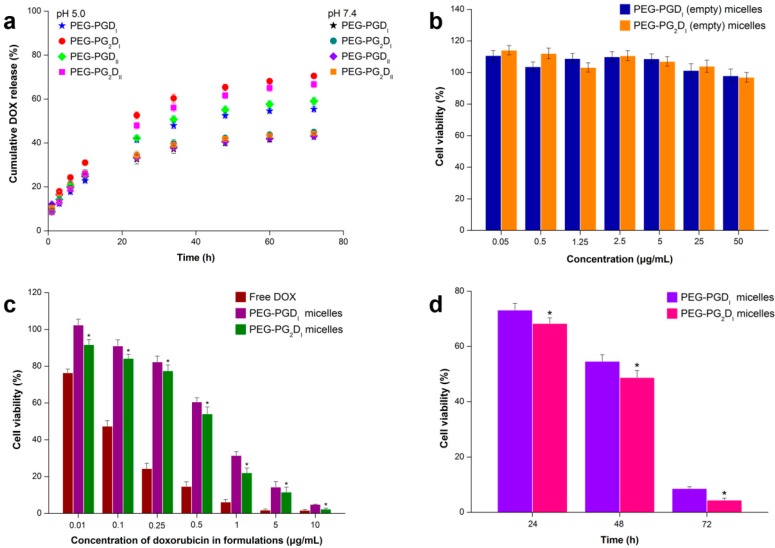
*In vitro* drug release and cytotoxicity assays. (**a**) *In vitro* DOX release profiles of the DOX-loaded polymer micelles at two different pH values (pH 5.0 and 7.4), Cytotoxicity of different concentrations of (**b**) empty polymer micelles and (**c**) Free DOX or DOX-loaded polymer micelles against HeLa cells by MTT assay, (**d**) Cytotoxicity of DOX-loaded polymer micelles against HeLa cells with different incubation times at the same drug concentration of 10 μg/mL.

*Note*: * *p* < 0.01.

The drug release behavior of the DOX-loaded LMs and YMs was investigated by dialysis diffusion at pH 5.0 and 7.4 [31]. The UV absorption spectra of DOX at different pH values (5.0, 7.4) were determined by UV spectrophotometry. Figure 5a shows DOX release patterns for PEG-PGD_I_, PEG-PGD_II_, PEG-PG_2_D_I_, and PEG-PG_2_D_II_.

A pH-dependent release pattern was observed in LMs and YMs with a 10%–26% higher DOX release at pH 5.0. The enhanced release was caused by acid-dependent breakage of hydrazone linkers [32]. All micelles demonstrated a similar release pattern at pH 7.4 with less than 45% DOX being released in 72 h. However, accelerated DOX release occurred at pH 5.0, and YMs showed a significantly higher release with almost 70% of the encapsulated drug being released in 72 h, which was 15% more than that of the LMs. The drug release pattern usually depends on a variety of factors like crystallinity, degradation rate, drug binding affinity, molecular weight, polymer composition, particle size, rate of hydration of the materials, surface characteristics and swelling ratio [33]. For micelles composed of drug-polymer conjugate, there are fewer chances for drugs to deposit on the surface of the micelles, which means the drug release rate of these micelles is primarily determined by the diffusion of the cleaved drug through the polymer matrix. This indicates that the hydrophilic blocks could to some extent control the release of the cargo as these hydrophilic blocks covering the micellar core functions like a retarding layer. For YMs, the branched drug-loading blocks that have larger volume increased the gap between the shell-forming PEG chains, decreased the density of PEG coverage, weakened the retarding layer thus facilitated drug release of YMs (Figure 5a). Moreover, to further explain the differences in release rates, we tested the solubilizing behavior of mPEG-poly(Glu-Hyd) and mPEG-poly(Glu-Hyd)_2_ with similar molecular weights in acidic media and found that mPEG-poly(Glu-Hyd)_2_ was more soluble (at pH 5.0) than mPEG-poly(Glu-Hyd). So, the differences in release rates could be explained by the relative hydrophilicity in acidic media of mPEG-poly(Glu-Hyd)_2_ after breakage of the hydrazone linker and the shorter chain lengths of the Y-shaped copolymers means less chain entanglement which accelerated the deformation of micelles. Compared with the established nanomedicine Doxil^®^, the formulation we prepared showed a more obvious acceleration in release rate under acidic environment, while pH has little influence on the release profile of Doxil^®^ [34]. However, Doxil^®^ presented better performance in sustained release [35]. These results indicate that YMs are able to release their cargoes promptly at the target site.

### 2.4. Cell Cytotoxicity Studies

As a drug carrier, biocompatibility is a primary concern, so the cytotoxicity of empty PEG-PGD_I_ and PEG-PG_2_D_I_ micelles was studied by MTT assay using HeLa cells. Figure 5b shows the cell viability after incubation for 24 h with polymeric micelles of empty PEG-PGD_I_ and PEG-PG_2_D_I_ at different concentrations. The results obtained showed that the cell viability of HeLa cells decreased as the concentration of micelles increased and the cell viability assay indicated that the empty PEG-PG_2_D_I_ copolymer was generally not very cytotoxic to HeLa cells at a concentration up to 50 μg/mL, demonstrating the excellent biocompatibility.

To investigate the antitumor activity of DOX-conjugated polymeric micelles, polymeric micelles incorporating doxorubicin were also examined using HeLa cells. The cell viability after incubation for 72 h with different concentrations of free DOX and DOX-conjugated micelles is shown in Figure 5c. In general, the viability of cells incubated with the two micelles decreased in a dose-dependent manner. Moreover, a lower cell viability (*p* < 0.01) of PEG-PG_2_D_I_ micelles compared with PEG-PGD_I_ micelles was observed at all concentrations. The IC50 value of free DOX, PEG-PG_2_D_I_ micelles and PEG-PGD_I_ micelles was 0.063, 0.517 and 0.673 μM, respectively. The time-dependent cytotoxicity of PEG-PGD_I_ and PEG-PG_2_D_I_ micelles in HeLa cells after incubation for 24 h, 48 h and 72 h with the same concentration (10 μM calculated by DOX) was compared and the results are summarized in Figure 5d. The cell viabilities decreased gradually as the incubation time increased and, at each time point, the PEG-PG_2_D_I_ micelles were more cytotoxic than PEG-PGD_I_ micelles. The mild acidic environments in tumor tissue [36] and in the endosomal lumen [37] can be used to facilitate drug release from the synthesized polymer-drug conjugates, and DOX may be taken up by cells via two different routes: part of the loaded DOX is released in tumor tissue and diffused into tumor cells, and the rest of the drug residing in micelles are endocyted and liberated within the endosomes. Since the cell viability is closely related to the amount of liberated DOX, the differences in cell viabilities could be attributed to the faster DOX release rates of PEG-PG_2_D micelles.

### 2.5. Cellular Uptake Study

**Figure 6:**
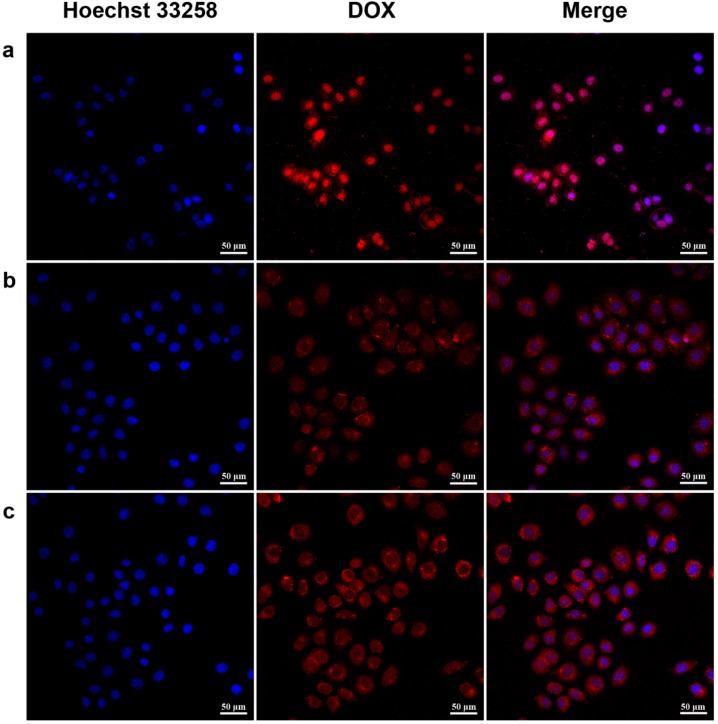
Uptake of DOX-loaded micelles by HeLa cells. Confocal laser microscopy (CLSM) images of HeLa cells incubated with DOX·HCl (**a**), DOX-conjugated PEG-PGD_I_ (**b**) and PEG-PG_2_D_I_ (**c**) micelle for 4 h with a DOX concentration of 10 μg/mL

Confocal laser microscopy (CLSM) was used to investigate the cellular uptake and intracellular distribution of free DOX, PEG-PGD and PEG-PG_2_D micelles in HeLa cells after a 4 h incubation. As shown in Figure 6, red fluorescence was observed in the cytosol, which indicated that the PEG-PG_2_D micelles and PEG-PGD micelles could be readily internalized by HeLa cells (Figure 6b,c). The relatively stronger fluorescence of PEG-PG_2_D micelles was caused by the accelerated DOX release after being internalized in the acidic lumen endosomes that was in accordance with the results of the drug release study. In Figure 6a, strong fluorescence of DOX was observed, which was due to the different cellular uptake mechanisms of free DOX and micelles. The free DOX could pass through the cell membrane by diffusion which is driven by a concentration gradient across the membrane, while micelles were taken up by endocytosis which is both time- and energy-consuming [38,39]. Since DOX is a compound that is highly permeable to cellular membrane, the difference between YMs and LMs in intracellular drug delivery was not clearly presented, which means a further study employing agents with poor cellular membrane permeability is warranted. According to the CLSM results, after entering the cell, free DOX can diffuse rapidly into the nuclei where they exert cytotoxic effects by inhibiting DNA replication [24]. However, DOX was liberated from the PEG-PGD or PEG-PG_2_D micelles conjugated with DOX in a controlled manner in the acidic endosomes, and then diffused into the cytosol before it finally entered the cell nuclei.

## 3. Experimental Section

### 3.1. Materials

α-Methoxyl-ω-amino poly(ethylene glycol) (mPEG-NH_2_, Mn: 5,000, polydispersity index: 1.02, Seebio Biotech, Inc. Co., Shanghai, P.R. China) and α-methoxyl-ω-N,N-bis(aminoethyl) poly(ethylene glycol) (mPEG-S-(CH_2_CH_2_NH_2_)_2_, Mn: 5,000, polydispersity index: 1.10, Seebio Biotech, Inc. Co.) were dried by azeotropic distillation with toluene; γ-benzyl-l-glutamate (BLG, Sichuan Tongsheng Amino Acid Ltd. Co., Deyang, China) and triphosgene (99%, GL Biochem, Shanghai, China) were dried before use; doxorubicin hydrochloride (DOX·HCL, Beijing Huafeng United Technology Ltd. Co., Beijing, China) was used as received. Triethylamine, hydrazine hydrate, anhydrous ethyl ether, methanol, dimethylsulfoxide (DMSO) and all other organic solvents used in the synthesis experiments were purchased from National Medicine Chemical Reagent Ltd. Co. (Shanghai, China). Other solvents were all dried before use. The water used in all experiments was double distilled. RPMI 1640 cell culture medium, fetal bovine serum (FBS), penicillin-streptomycin, trypsin, Dulbecco’s phosphate buffered saline (DPBS), dimethyl sulfoxide (DMSO) and 3-(4,5-dimethylthiazol-2-yl)-2,5-diphenyltetrazolium bromide (MTT) were purchased from Gibco Company (New York, NY, USA).

### 3.2. Synthesis of γ-benzyl-L-glutamate-N-carboxy anhydride

γ-Benzyl-l-glutamate-*N*-carboxy anhydride (BLG-NCA) was prepared according to the published method [40] with some modifications. Briefly, BLG (8.0 g, 54.4 mmol) was suspended in dry THF (80 mL) and heated to 55 °C. After 30 min, a solution of triphosgene (4.0 g, 13.5 mmol) in anhydrous THF (40 mL) was added dropwise under stirring, and the reaction was conducted under argon atmosphere until the mixture became transparent (about 2 h). The solvent was removed by rotary evaporation under reduced pressure and the crude product was washed with anhydrous hexane to remove residual triphosgene. Then, the crude crystals of BLG-NCA were recrystallized three times in a mixture of EtAc, THF and petroleum ether at room temperature to obtain white crystalline needles.

### 3.3. Synthesis of Methoxyl Poly (ethylene glycol)-Poly (γ-benzyl-l-glutamate) Diblock and AB_2_ Y-Shaped 3-Miktoarm Star-Block Copolymers

mPEG-Poly(γ-benzyl-l-glutamate)(PBLG) diblock and mPEG-(PBLG)_2_ 3-miktoarm star copolymer were synthesized by ring-opening polymerization (ROP) of BLG-NCA initiated by mPEG-NH_2_ or mPEG-S-(CH_2_CH_2_NH_2_)_2_ in anhydrous DCM with different feed ratios. The polymerization reaction was carried out with stirring under argon atmosphere at 25 °C for 72 h. The mixture was evaporated under reduced pressure to reduce the volume, and then precipitated twice with anhydrous ethyl ether. The precipitate was collected by filtration, dissolved in THF and dialyzed (cellulose ester, MWCO: 3500) against deionized water. Finally, the fluffy white powder was collected by freeze-drying.

### 3.4. Synthesis of Methoxyl Poly (ethylene glycol)-Poly (glutamate-hydrazone-doxorubicin)_2_ Polymer

The benzyl esters of mPEG-PBLG diblock copolymer and mPEG-(PBLG)_2_ miktoarm-star copolymer were substituted with hydrazide (Hyd) by an ester-amide exchange (EAE) aminolysis reaction to obtain mPEG-poly(Glu-Hyd) and mPEG-poly(Glu-Hyd)_2_ polymers [24]. Excess hydrazine hydrate (20 times the number of BLG repeating units) was reacted with mPEG-PBLG or mPEG-(PBLG)_2_ in DMSO at 40 °C for 24 h. The polymer products were purified by repetitive ether precipitation, dialysed against deionized water (MWCO 7 kDa), and collected by freeze-drying. Then, the neutralized DOX was conjugated with the hydrazide groups of mPEG-poly(Glu-Hyd) or mPEG-poly(Glu-Hyd)_2 _in DMSO with a 1:1 molar ratio at 30 °C for 2 days. The products were purified by extensive dialysis against methanol for 2 days and then dialysis against deionized water for 1 day to remove the unbound DOX. The final product was collected by freeze-drying.

### 3.5. Material Characterization

Proton nuclear magnetic resonance (^1^H-NMR) measurements were carried out on all samples using a Bruker DMX 600 spectrometer at 600M (Billerica, MA, USA). Fourier transform infrared spectroscopy (FT-IR) spectra were recorded on a Bruker Tensor 27 spectrometer using potassium bromide (KBr) disks. Polydispersity indices (PDI) of copolymers were determined by a gel permeation chromatography (GPC) system performed on a Waters 1515 GPC instrument (Waters Corp, Milford, MA) equipped with three Styragel columns (Waters Corp; 10^5^, 10^4^, and 10^3^ Å) in tandem and a 2414 differential refractive index detector. DMF was used as the eluent at a flow rate of 1.0 mL/min. The column temperature was set at 35 °C. The critical aggregation concentration (CAC) of the drug-conjugated copolymers was measured using a microplate reader (Bio Rad 680, Bio Rad, Hercules, CA, USA) with pyrene as probe [17] by recording excitation spectra from 300 nm to 350 nm at fixed emission wavelength (390 nm). The excitation of DOX was excluded by using DOX solutions as blanket, and the differences of fluorescent intensity between DOX solution and drug-conjugated copolymer solutions at various concentrations were used to calculate CACs.

### 3.6. Preparation and Characterization of the pH-Sensitive Polymeric Micelles

DOX-conjugated micelles were prepared by first dissolving the polymers in DMSO, and then dialyzing in a dialysis tube (MWCO: 14,000) against phosphate buffer saline (pH 7.4, 10 mM) for 24 h and deionized water for 4 h to eliminate salts. The prepared micelle solution was passed through a 0.45 μm filter to remove any precipitate. The particle size and distribution of the micelles were determined by dynamic laser light scattering (DLS) with a NICOMP^TM^ 380 Submicron Particle Sizer (Particle Sizing Systems, Santa Barbara, CA, USA) at room temperature. A laser beam at a wavelength of 632.8 nm was used, and the scattering angle was fixed at 90° when measurements were conducted. DOX loading content was determined using ultraviolet-visible spectrophotometry (UV-Vis, absorbance at 485 nm, T6 PERSEE, Beijing, China) by first cleaving DOX from the polymer backbone by 0.1 N HCl. The micelle morphology was examined by transmission electron microscopy (TEM H-600, Hitachi, Tokyo, Japan) with an accelerating voltage of 100 kV [41].

### 3.7. In Vitro Release Studies

The pH-responsive DOX release behavior of the micelles was investigated by dialysis diffusion [7]. Briefly, all micelles conjugating DOX were dispersed in 3 mL of medium (phosphate buffer (10 mM, pH 7.4) or acetate buffer (10 mM, pH 5.0)) and transferred to a group of three pre-swelled dialysis tubes (cellulose ester, MWCO: 14,000). Subsequently, the tubes were immersed in 20 mL of the same medium and placed in a horizontal water bath shaker maintained at 37 ± 0.5 °C with a shaking speed of 100 rpm. Then, 3 mL of release medium was replaced with fresh medium at predetermined intervals, and the samples taken were analyzed by UV-spectrophotometry at 485 nm to determine the DOX release. All data were obtained in triplicate using parallel experiments and were expressed as mean values.

### 3.8. In Vitro Cytotoxicity Assay

Human cervical carcinoma (HeLa) cells (provided by the Cell Center of the Chinese Medical University) were cultured in RPMI 1640 medium, supplemented with 10% (v/v) fetal bovine serum (FBS) and 1% (v/v) penicillin-streptomycin (100 U/mL penicillin and 100 μg/mL streptomycin) at 37 °C in a humidified atmosphere with 5% CO_2_. The cells were harvested with 0.25% trypsin and rinsed. The resulting cell suspensions were used in the subsequent experiments.

The cytotoxicity of the empty polymeric micelles, free DOX, and DOX-conjugated micelles against HeLa cells was evaluated using an MTT (3-(4,5-dimethylthiazol-2-yl)-2,5-diphenyltetrazolium bromide) cell proliferation assay[42]. Briefly, HeLa cells were seeded at a density of 3,000 cells per well in 96-well plates for the incubation and attachment of the cells. After 24 h, the growth medium was removed and replaced with 100 μL fresh medium containing serial dilutions of the samples (n = 5). Cells were then incubated at 37 °C, 5% CO_2_, for 72 h. After predetermined periods of incubation, MTT solution in PBS (20 μL, 5 mg/mL) was added to the sample-treated cells and incubated for the next 4 h. The culture medium was replaced with 100 μL DMSO, followed by shaking for 10 min to dissolve the blue formazan crystals formed. The optical density (OD) of each well was then measured at 490 nm using a Multiscan Spectrum microplate reader (Thermo Fisher Scientific, Vantaa, Finland). Cells cultured without micelle solution were used as the blank control. Cell viability was calculated according to following equation [43]:

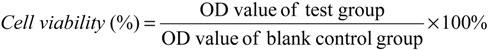
(1)


### 3.9. Confocal Analysis

HeLa cells were seeded in a 6-well plate and incubated for attachment. After seeding for 24 h, the medium was aspirated, and fresh medium containing free DOX or DOX-loading micelles was added into the wells (DOX concentration: 10 μg/mL). After incubation for 4 h, cells were washed with phosphate-buffered saline (pH 7.4, 0.1 M) twice for 2 min each. Next, cells were fixed with 4% formaldehyde, washed with phosphate-buffered saline. Then, the cells were stained with Hoechst 33258 for 20 min and washed with PBS twice for 2 min each. These cells were observed under a confocal laser scanning microscope (Zeiss LSM 710 META, Jena, Germany) with 488nm argon laser excitation.

## 4. Conclusions

In summary, linear and Y-shaped copolymers with DOX conjugated by an ester-amide exchange (EAE) aminolysis reaction were synthesized and characterized. Micelles with core/shell structures were prepared by self-assembling of these copolymers in aqueous milieu. For YMs and LMs with similar DLC, smaller diameters were observed in YMs due to the introduction of another drug-loading chain that shortened the lengths of the hydrophobic blocks. Also, a shorter drug-loading chain in the Y-shaped copolymers could facilitate drug release due to more loose PEG coverage and less chain entanglement that further achieves prompt drug release under acidic conditions. YMs based on the newly synthesized copolymers showed a higher cytotoxicity than that of the traditional LMs, while micelles composed of non drug-conjugated polymers showed almost no tumor-suppressive effect against HeLa cells up to a concentration of 50 μg/mL. These results demonstrate that poly aminoacid-based Y-shaped copolymers can be used as effective building blocks in the construction of effective drug delivery systems and further *in vivo* studies are warranted.

## Supplementary Materials

Supplementary materials can be accessed at: http://www.mdpi.com/1420-3049/19/8/11915/s1.

## Supplementary Files

Supplementary File 1
