# Supplementary material for: Self-Assembled Micelles Composed of Doxorubicin Conjugated Y-Shaped PEG-Poly(glutamic acid)2 Copolymers via Hydrazone Linkers
Source: Molecules. 2014 Aug 11;19(8):11915–32. doi: 10.3390/molecules190811915 (PMC6271086; doi:10.3390/molecules190811915)

Supplementary File

Self-Assembled Micelles Composed of Doxorubicin Conjugated Y-Shaped PEG-Poly (glutamic acid)$_2$ Copolymers via Hydrazone Linker

Figure S1. ^{1}H-NMR spectra of PEG_{5k}-$PBLG_{5.7k}$ (**a**); PEG_{5k}-$PBLG_{13.6k}$ (**b**); PEG_{5k}-$(PBLG_{5.7k})_2$ (**c**) and PEG_{5k}-$(PBLG_{6.8k})_2$ (**d**) copolymer in $CDCl_3$.

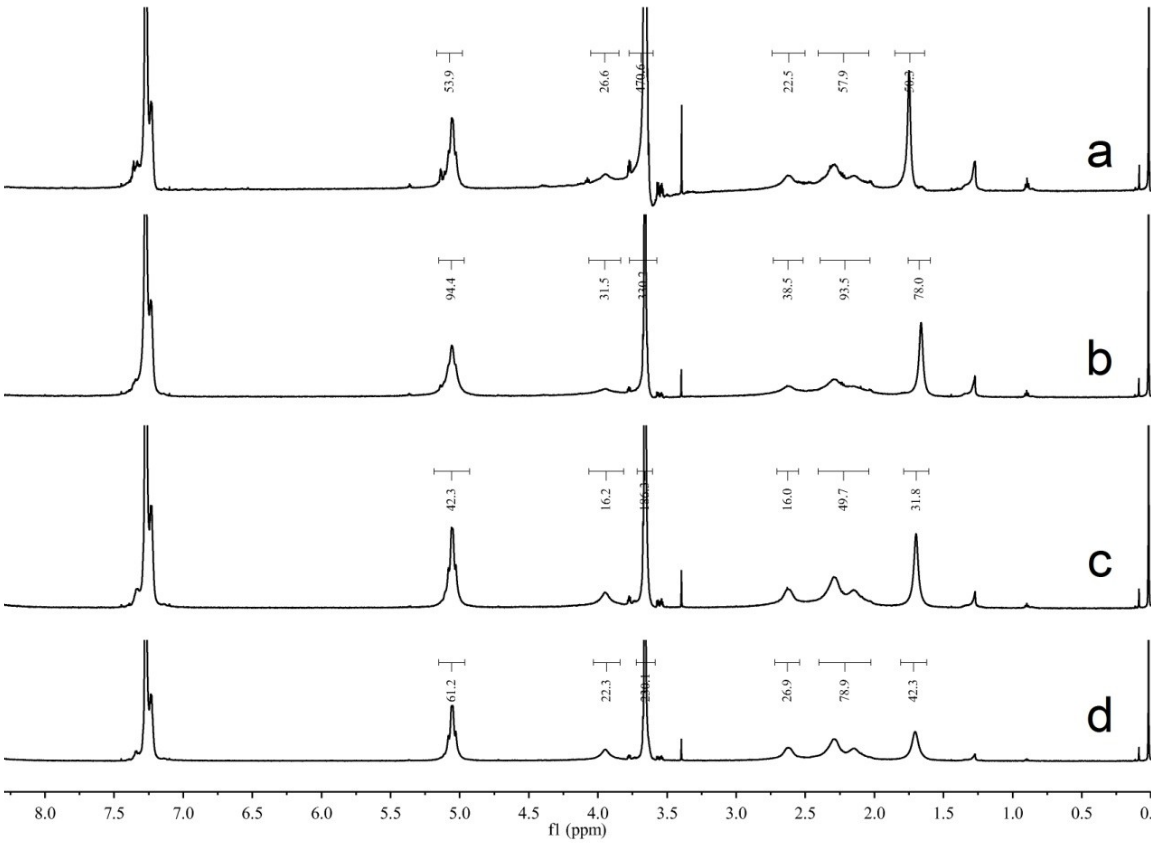

Figure S2. ^{1}H-NMR spectra of PEG-p(Glu_{26}-Hyd) (**a**), PEG-p(Glu_{62}-Hyd) (**b**), PEG-p(Glu_{26}-$Hyd)_2$ (**c**) and PEG-p(Glu_{31}-$Hyd)_2$ (**d**) polymer in CF_3COOD/DMSO-d_6.

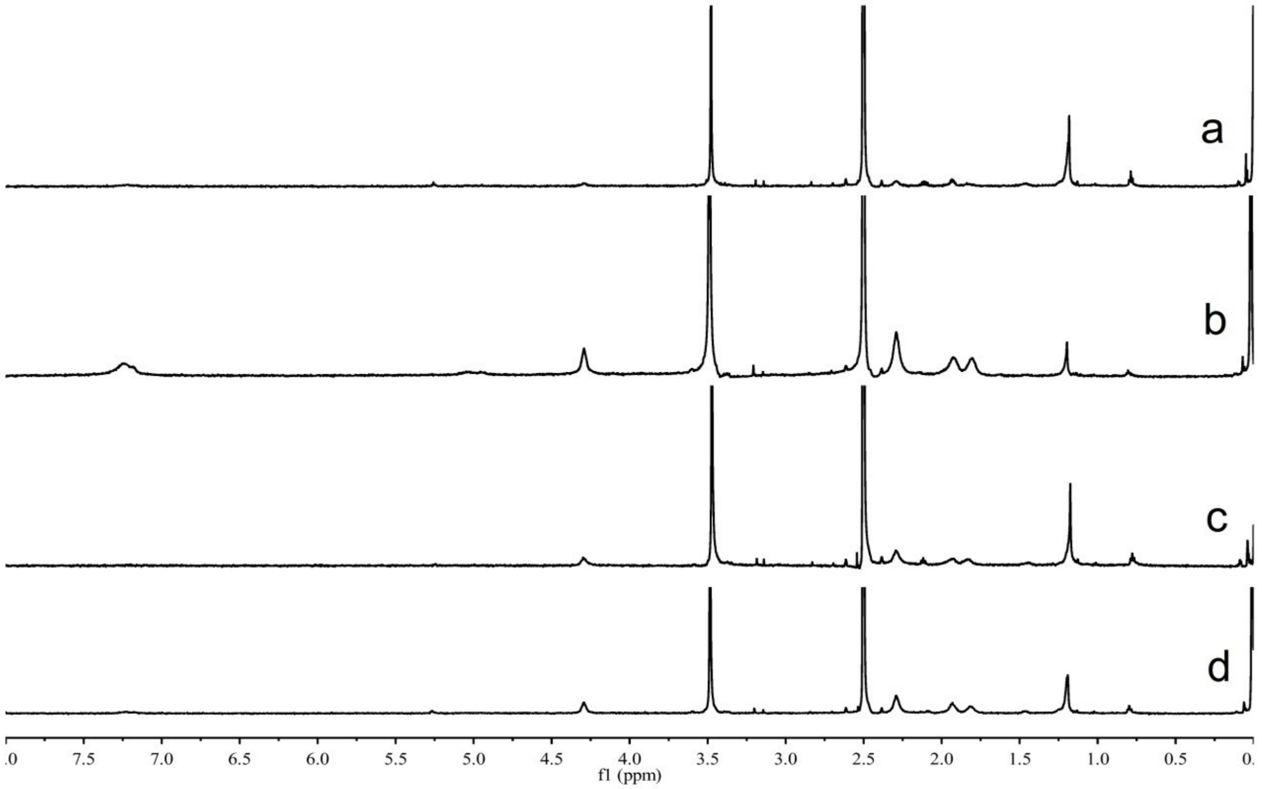

Figure S3. DMF gel permeation chromatography traces of PEG-NH_2 (**a**); PEG-S-$(CH_2CH_2NH_2)_2$ (**b**); PEG_{5k}-$PBLG_{5.7k}$ (**c**); PEG_{5k}-$(PBLG_{5.7k})_2$ (**d**); PEG_{5k}-$PBLG_{13.6k}$ (**e**) and PEG_{5k}-$(PBLG_{6.8k})_2$ (**f**) copolymers.

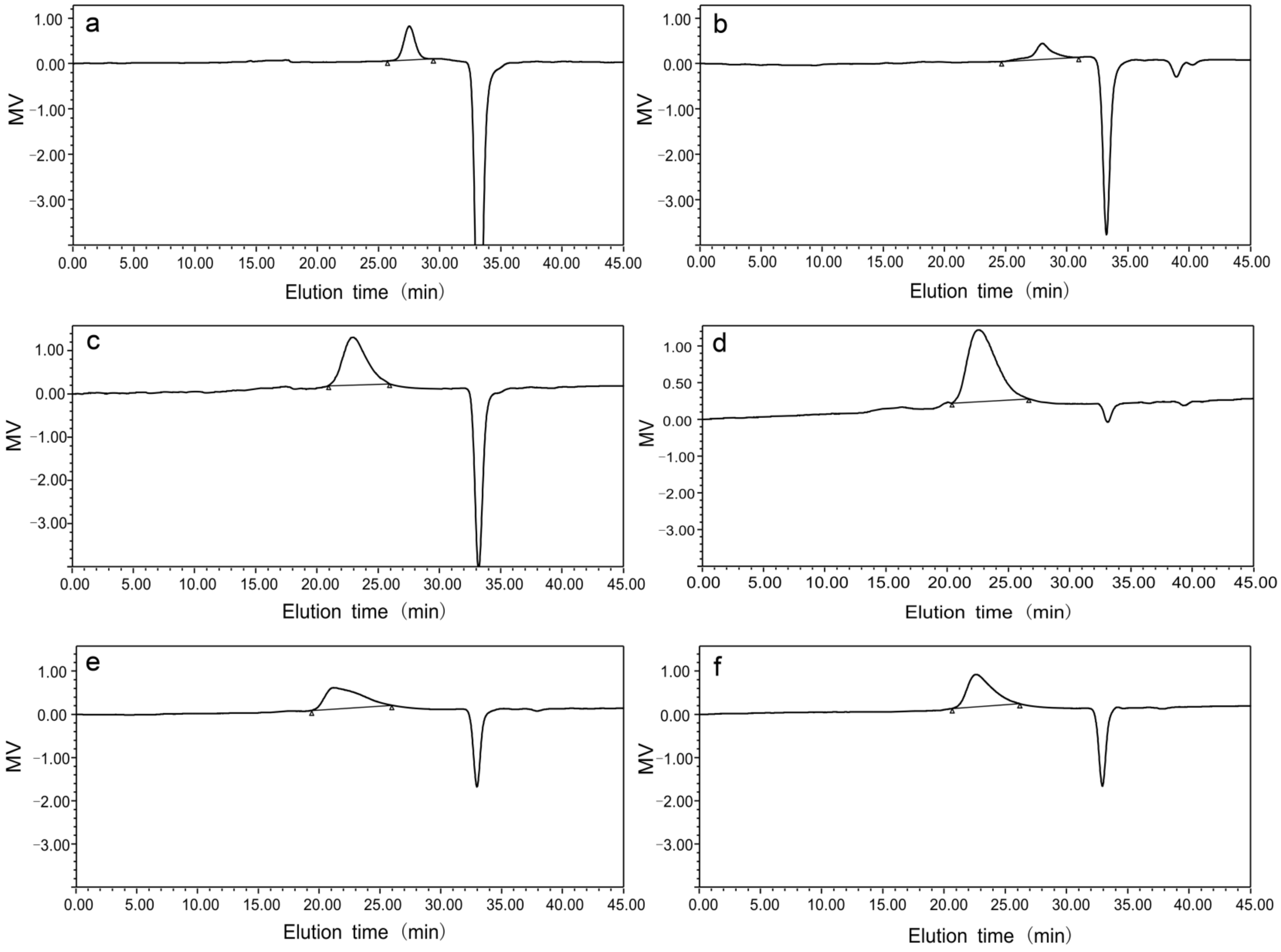

Supplement: Supplementary File 1 [file molecules-19-11915-s001.pdf]
